# Efficacy Comparison of Five Different Acupuncture Methods on Pain, Stiffness, and Function in Osteoarthritis of the Knee: A Network Meta-Analysis

**DOI:** 10.1155/2018/1638904

**Published:** 2018-11-01

**Authors:** Shaowei Li, Pingjin Xie, Zhenghui Liang, Weihan Huang, Zhanhui Huang, Jinming Ou, Zhiyong Lin, Shengting Chai

**Affiliations:** ^1^Guangzhou University of Chinese Medicine, Guangzhou 510405, Guangdong, China; ^2^Department of Orthopaedics, the Affiliated Orthopaedics and Trauma Hospital of Guangzhou University of Chinese Medicine, Guangzhou 510240, Guangdong, China

## Abstract

The principal objective of this present study was to compare the effects of different acupuncture methods on pain, stiffness, and physical function for osteoarthritis of the knee by the pairwise and network meta-analysis (NMA). A network meta-analysis of randomized controlled trials (RCTs) was searched from three English databases and one Chinese database until January 2018. A pairwise meta-analysis was performed with a random effects model. Then we carried out the NMA within a Bayesian framework. Mean difference (MD) and its 95% confidence interval (CI) were calculated by R 3.4.1, Stata 14.0, and RevMan 5.3 software to assess the relief of pain, the effectiveness for stiffness, and physical function recovery. Node-splitting method was used to calculate the inconsistency. Rank probabilities were assessed and clustered by the surface under the cumulative ranking curve (SUCRA). 16 trials mostly researched short-term effectiveness and showed that fire needle and electro-acupuncture were statistically significant to decrease WOMAC pain and physical function scores when compared with other treatments, but there was no significant difference in stiffness calculations. Our NMA demonstrated that acupuncture with heat pain or electrical stimulation might be suggested as the better choices in all acupuncture methods to osteoarthritis of the knee.

## 1. Introduction

Osteoarthritis (OA) refers to a clinical syndrome of joint pain accompanied by varied degrees of functional limitation and reduced quality of life, which is common degenerative disease of knee joint in elderly patients [1, 2]. It is reported that approximately 6% adults whose age above 30 years have suffered from symptomatic OA, and 80% of people over the age of 70 years have undergone the harm brought by Knee OA [3, 4]. Largely attributable to the effects of disability, comorbid disease, and the expense of treatment, the extremely high economic burden of knee osteoarthritis (KOA) also remains a huge healthcare challenge in a continuous process of aging society [5, 6].

There are many methods for clinical treatment of KOA. The first-line drugs are nonsteroidal anti-inflammatory drugs (NSAIDs), but it cannot slow down the progress of KOA, which even has been linked to an elevated risk for cardiovascular and gastrointestinal adverse events and renal toxicity in recent studies [7]. So many people highlight the nonpharmacological modalities, such as good education and self-management, muscle strengthening and water-based exercises, weight reduction, walking aids, knee braces, and acupuncture in OARSI evidence-based and expert consensus guidelines [8].

**Figure 1:**
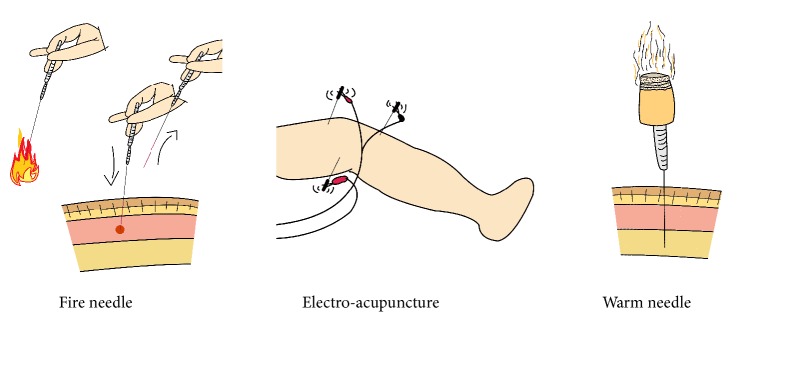
A diagram of fire needle, electro-acupuncture, and warm needle.

Among the above-mentioned therapies, acupuncture therapy consisting of “needle" and “moxibustion" is one of the important components of oriental medicine. More research [9–18] continues to show that acupuncture, electro-acupuncture, fire needle, warm needle (Figure 1), and even sham needle for patients with KOA all play a positive role in pain, stiffness, and physical function in varying degrees. Based on an evolutionary theory, new studies have demonstrated that it can effectively modulate the pain control system to improve daily living capability [19].

Developed from the traditional manual acupuncture, electro-acupuncture (EA) is a therapy that passes a microcurrent wave of human bioelectricity through the needle after de qi during acupuncture therapy. Recent advances in EA may permit activation of specific neuronal networks to prevent organ damage in inflammatory and infectious disorders, and many studies have manifested that EA is a good opportunity to significantly reduce pain and make lower risk of adverse reactions to improve the patients' stiffness and physical function with osteoarthritis of the knee, through an evidence-based process [20, 21].

Warm needling (WN) [22] is a combination therapy of acupuncture and moxibustion by burning moxa in the needle tail with acupoint stimulation and the effects of warming up. Somewhat differently, the fire needle (FN) has the property of high temperature resistance, and needle retention time of the fire needle is less than that of original needle and warm needle [23]. However, they are both in varied degrees of impact on relieving physical pain and delaying inflammation development process such as Herpes Zoster [24], Rheumatoid Arthritis [25], and KOA [23].

Although many studies have assessed the significant effects of different types of acupuncture treatment on KOA, the conclusions are inconsistent. Especially due to the lack of direct comparison of randomized controlled trials between different treatments, it is not conducive to the choice of clinical application and the implementation of the best treatment. Therefore, we compare the effectiveness of different types of acupuncture in alleviating the pain, stiffness, and functional activity of osteoarthritis of the knee joint by using a network meta-analysis method and order the therapeutic measures according to the curative effect in order to provide comprehensive and reliable evidence-based medical evidence for clinical practice.

## 2. Materials and Methods

### 2.1. Inclusion Criteria

(1) Studies were randomized controlled trials (RCTs). (2) Patients were diagnosed with knee osteoarthritis. (3) Interventions were compared between common manual acupuncture, electro-acupuncture, fire needle, warm needle, placebo, sham needle, or education. (4) The outcome indicators were Western Ontario and McMaster Osteoarthritis Index (WOMAC) pain, stiffness and physical function scores.

### 2.2. Exclusion Criteria

Exclusion criteria were (1) repeated articles; (2) ambiguous diagnostic criteria and efficacy observation indicators; (3) abstract articles, review articles, and animal experiments; (4) the included interventions which could not form a network meta-analysis.

### 2.3. Data Sources and Search Strategy

The PubMed (https://www.ncbi.nlm.nih.gov/pubmed/), the Cochrane Library (http://www.cochranelibrary.com/), the EMBASE (http://www.embase.com/), and the CNKI databases (http://www.cnki.net/) from their inception to January 2018 were searched in order to collect RCTs related to the relationship between different acupunctures and KOA. The search used the combination of a subject word and a free word: osteoarthritis, acupuncture, electro-acupuncture, fire needle, warm needle, WOMAC and randomized trails, etc.

### 2.4. Data Extraction and Quality Assessment

According to the exclusion criteria, two evaluators (SW Li and ZH Liang) independently screened all trials for inclusion and extracted the data for cross-check. If there was some disagreement about the details between the two reviewers, a final decision would be solicited by the third evaluators (PJ XIE). Data extraction contents included (1) basic information included in the study, such as the study author and year of publication; (2) baseline table of the treatment group and control group which included the sample size and gender of each group; (3) intervention measures which were specific details, follow-up time, etc.; (4) outcomes which involved WOMAC pain, WOMAC stiffness, and WOMAC physical function; (5) risks of bias assessment of eligible studies that were evaluated according to the Cochrane Collaboration Handbook (http://www.cochrane.de).

### 2.5. Data Synthesis and Statistical Analysis

All data analyses were conducted by Review Manager Software Version 5.3 Stata Version 14.0 and R Software Version 3.4.1. First of all, a pairwise meta-analysis was performed by the Rebecca DerSimonian and Nan Laird method [32]. Secondly, a network meta-analysis within the Bayesian framework and the Markov Chain Monte Carlo (MCMC) model was produced by using the GEMTC package of the R 3.4.1 software from all eligible randomized controlled trials. A random effects model was selected because of heterogeneity to assess the effectiveness of acupuncture for KOA. Mean difference (MD) and its 95% confidence interval (CI) were used to present the continuous outcomes such as changes in Western Ontario and McMaster Universities Osteoarthritis Index (WOMAC) of pain, stiffness, and function scores, by estimating the final measurement values with change from baseline points. Clinicians could judge whether there was a difference in the efficacy of the two treatments by considering the upper and lower limits of 95% confidence intervals (95% CI) for a study. If a study of the 95% CI did not contain value 0, it was generally believed that there were differences in the efficacy between the two treatments.

Finally, the inconsistency test was performed using the node-split model. The node-splitting plot could be used to determine the clinical authenticity of NMA by its* P* value. If there was no statistical difference (P>0.05), the consistency model was used for analysis; otherwise, the inconsistency would be reported due to the statistical significance's comparison of direct and indirect evidence (P<0.05) [33]. At the same time, we should provide suggestions for clinical treatment intuitively under simple clinical factor or multiple clinical factors. In this case, the effects of different acupuncture methods to reduce the scores of WOMAC would be reflected by the surface under the resurfacing curve (SUCRA), the plot area estimation of the efficacy of each intervention method, and the clustered ranking plot for all outcomes [34, 35].

We carried out sensitivity analyses by excluding one study at a time to evaluate the influence of single study on the overall results in RevMan Software 5.3. The publication bias was assessed by the Egger regression asymmetry test in Stata 14.0.* P* value more than 0.05 was considered as significant [36]. The above two statistical methods could be used to assess the stability of results from direct comparisons to prove that the NMA results were reliable clinically. Diagram plots were drawn by Stata 14.0, such as the network diagram, the cumulative contribution plots, and the publication bias graphs. The clinical significance of the networks was to explain which measures were directly compared and which were indirectly compared. Meanwhile, the cumulative contribution plots and the publication bias graphs could further verify whether the evidence of results was reliable in clinical practice.

## 3. Results

### 3.1. Literature and Patient Characteristics

**Figure 2:**
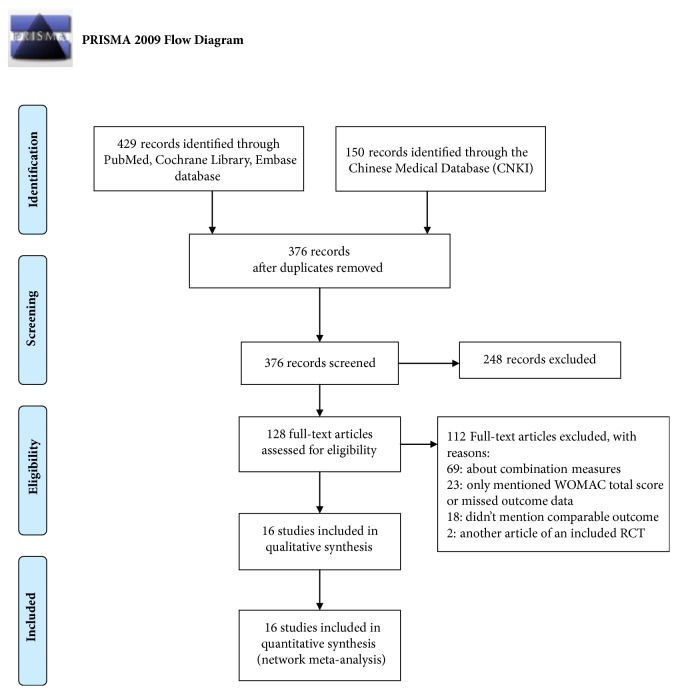
PRISMA 2009 flow chart of the study searching process.

The literature screening process and results could be seen in (Figure 2). According to the screening sequence of PRISMA 2009 flow diagram, the search strategy retrieved 579 related records. After screening carefully, we picked up 16 eligible articles including a total of 2065 KOA patients. The experimental group had 1891 people, and the control group had 1764 people, each from the United States, Canada, Spain, Thailand, Germany, and China. The baselines for gender, age, sample size, and condition were basically the same among the studies. What is more, these RCTs were conducted between 1994 and 2017 with a maximum following-up time of more than 2 years. The treatment group had common manual acupuncture, electro-acupuncture, fire needle, etc. The control group were sham needle, no intervention, and education. There were 14 two-armed and 2 three-armed experiments in the study (Table 1).

### 3.2. Methodological Quality Assessment

**Figure 3:**
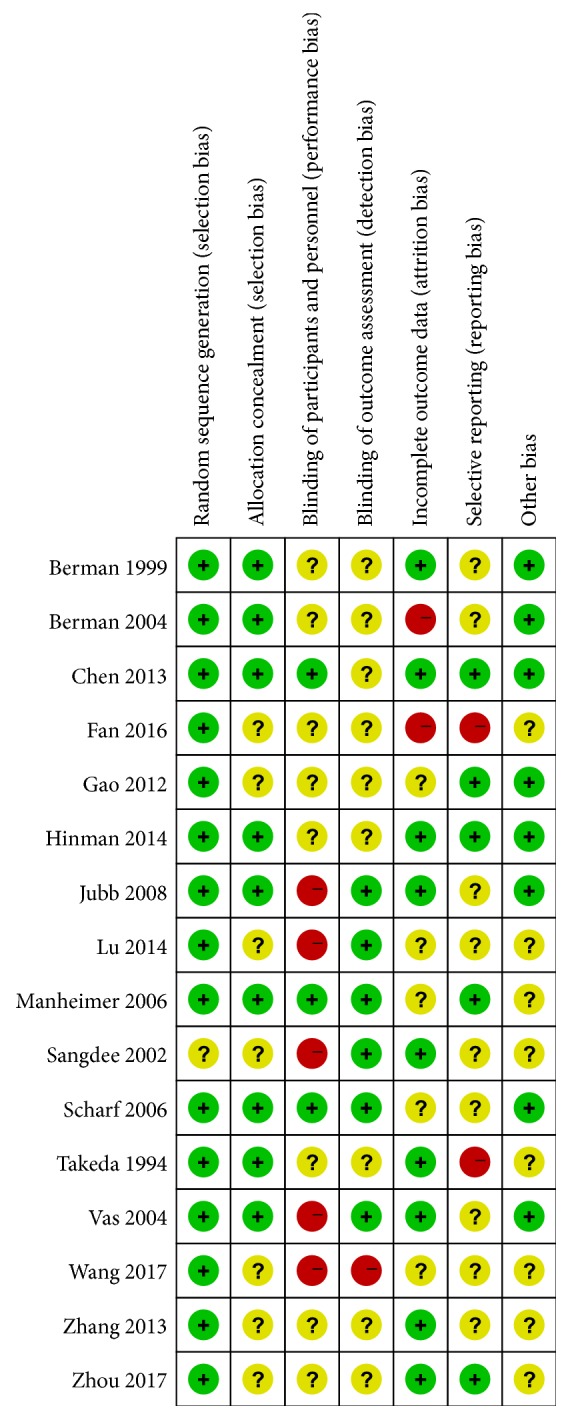
Diagram of bias risk.

Risks of bias assessment of eligible studies illustrated that the number of participants randomized was clearly stated in all studies (Figure 3). Except one report [9], all clearly reported using suitable randomization methods, such as opaque envelope. And nine studies [10–12, 14, 26–29, 31] reported the appropriate methods for concealing treatment allocation. Only three studies [10, 14, 29] completely met the blind criterion. In addition to five studies [9, 12, 13, 17, 31] failing to mention the blind method, the rest partially met the blind requirements. All studies but four [15–17, 30] reported whether there were any losses to follow up. Group baseline characteristics appeared comparable in all trials.

### 3.3. Results of Conventional Pairwise Comparison

With a random effects model, the total twenty-five direct comparisons were produced in a conventional pairwise meta-analysis. When the 95% CI upper and lower limits of a study were all less than value 0, experimental factors in treatment group measured by the adjusted mean change from baseline could be considered beneficial if the investigator's event was a beneficial event. A subgroup analysis showed that acupuncture and electro-acupuncture were statistically significant to relieve pain and improve function in KOA patients over sham needle control groups (MD = -1.16, 95%CI [-1.51, -0.82]; MD = -3.34, 95% CI [-4.68,-1.99]). Meanwhile, acupuncture, electro-acupuncture, and warm needle were better than education and no intervention control (MD = -2.09, 95%CI [-2.15, -2.03]; MD = -6.60, 95%CI [-6.97, -6.22]). In terms of stiffness, acupuncture, electro-acupuncture, and warm needle did well in relieving the rigidity sense (MD = -0.687, 95%CI [-1.264, -0.110]). What is more, the fire needle control might be more effective than the electro-acupuncture in alleviating knee pain and stiff sense and improving physical function (MD = -2.57, 95%CI [-3.67, -1.47]; MD = -1.80, 95% CI [-3.11,-0.49]; MD = -4.95, 95% CI [-5.63,-4.27]) (Tables 2, 4, S1, S2 and S3).

### 3.4. NMA of Different Acupuncture Interventions

**Figure 4:**
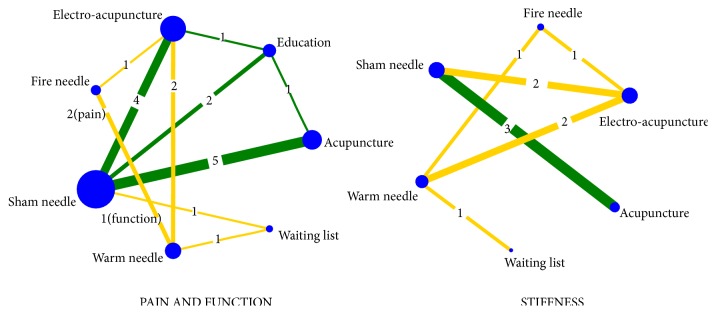
Network comparisons of acupuncture methods for KOA.

**Figure 5:**
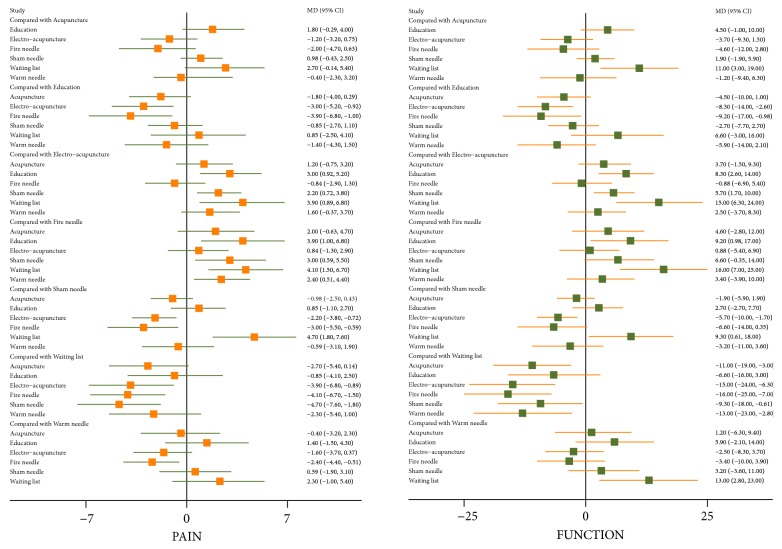
Forest plots of NMA with respect to acupuncture, electro-acupuncture, fire needle, warm needle, waiting list, sham needle, and education in (WOMAC) pain and physical function scores.

**Figure 6:**
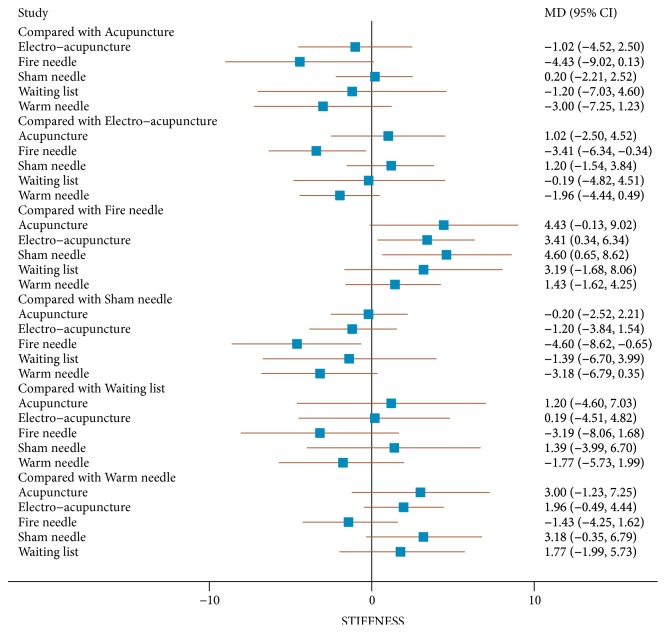
The forest plot of NMA with respect to acupuncture, electro-acupuncture, fire needle, warm needle, waiting list, sham needle, and education in (WOMAC) stiffness scores.

The direct comparison of all measures constituted a network diagram. The thickness of the line and the size of the circle indicated the number of the two groups of studies and the total sample size of their treatment measures. The lines were using different coloured edges on behalf of the different risk of allocation concealment. Green meant low risk, Yellow meant unclear risk, and Red meant high risk (Figure 4). Both direct and indirect evidence were generated by change scores from baseline in NMA. When the iterations reached 50000, the PSRF were all close to 1, indicating a good convergence, so we used the consistency model for analysis. And Tables 3 and 4 and Figures 5 and 6 included the results of the NMA for the outcome measure of pain, stiffness, and function for all trials. In a forest plot for our study, if the horizontal line of 95% CI did not intersect with the invalid vertical line, it was considered to have a clinical significance. In other words, a study of the 95% CI was exclusive of value 0 which stated clearly that the comparison between treatment measures was meaningful. Especially when the horizontal line fell to the left of the invalid line, if the indicator studied by the researcher was not an adverse event, the experimental factor would be treated as a favourable factor. Under the circumstances, when MD was more than value 0 in this NMA, it indicated that the therapeutic effect of the columns was worse than that of the rows. Instead (MD<0), it meant that the treatment in columns was more effective than that of the rows. On the hand of pain outcome, patients with fire needle indicated that it could improve pain outcome over education, sham needle, warm needle, and waiting list group (MD = -3.90, 95%CI [-6.80, -1.00]; MD = -3.00, 95%CI [-5.50,-0.59]; MD = -2.40, 95%CI [-4.40, -0.51]; MD = -4.10, 95%CI [-6.70, -1.50]). In addition, compared with education, sham needle, and waiting list group, patients with electro-acupuncture also significantly exhibited the superiority on reducing pain scores (MD = -3.00, 95%CI [-5.20, -0.92]; MD = -2.20, 95%CI [-3.80, -0.72]; MD = -3.90, 95%CI [-6.80, -0.89]). In our study, we found that sham needle was more effective than no treatment group (MD = -4.70, 95%CI [-7.60, -1.80]). On the other hand, the results of physical function outcome demonstrated that electro-acupuncture and fire needle also powerfully evidenced their effect to improve physical function. And patients with warm needle and acupuncture could have better physical function due to knee osteoarthritis, compared with no intervention, education, and acupuncture groups (MD = -13.00, 95%CI [-23.00, -2.80]; MD = -11.00, 95%CI [-19.00, -3.00]). NMA of stiffness calculations showed that fire needle was more useful to alleviate the stiff sense than electro-acupuncture and sham needle (MD = -4.60, 95%CI [-8.62, -0.65], MD = -3.41, 95%CI [-6.34, -0.34]).

### 3.5. Ranking Probability and Cluster Analysis with SUCRA

**Figure 7:**
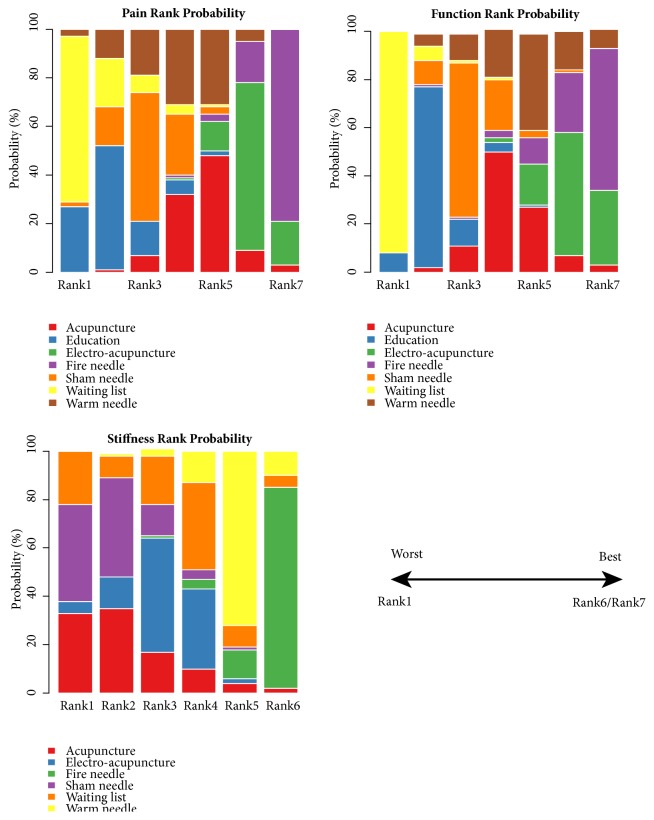
Probability ranking plots of acupuncture, electro-acupuncture, fire needle, warm needle, waiting list, sham needle, and education in (WOMAC) pain, stiffness, and physical function scores.

**Figure 8:**
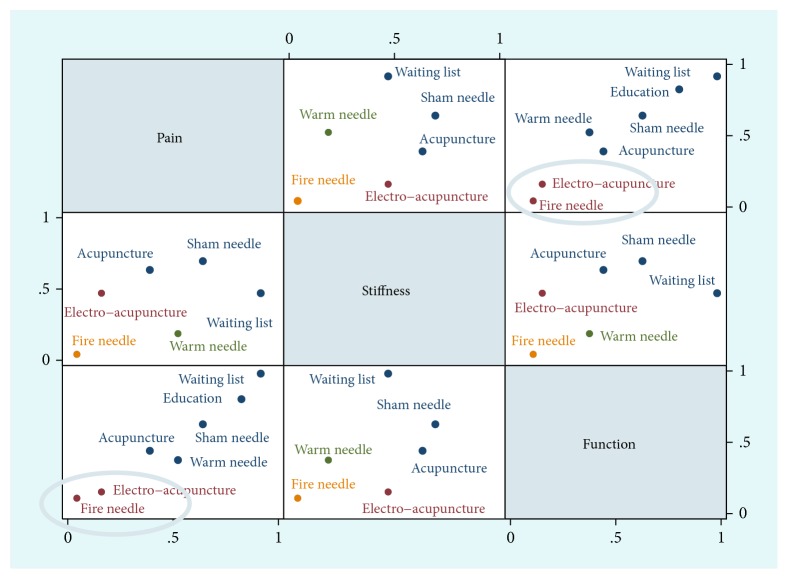
Clustering analysis plots of acupuncture, electro-acupuncture, fire needle, warm needle, waiting list, sham needle, and education in (WOMAC) pain, stiffness, and physical function scores.

The ranking probabilities of each intervention with respect to each endpoint and colour occupancy area were presented in (Figure 7, Table 5). The inferiority to superiority was from rank1 to rank7 (or stiffness was up to rank6). Especially when a measure was taking up more areas in rank1 plot, it meant worse effects due to the final value changing from baseline measurement. However, a larger portion of rank7 represented better effects, which could better occur to KOA patients' less pain, stiffness, and dysfunction scores. According to cumulative probability of being the most effective intervention, the results showed that fire needle was the best. But clinically, there were usually several factors to consider when recommending interventions. In our study, the factors contained pain, stiffness, and physical function. To solve this problem, we could use a clustering analysis based on cumulative probability of being the most effective intervention. The different colours represented the estimated clusters, and then the treatment was grouped according to their similarity in the three outcomes. According to a cluster analysis, the scatter plot about pain reduction, stiffness alleviation, and physical function improvement was presented in (Figure 8). The SUCRA value of two endpoints was used to differentiate and cluster the above KOA interventions. Through synthetical consideration, fire needle and electro-acupuncture could be an optimization method.

### 3.6. Inconsistency Test between Direct and Indirect Evidences

**Figure 9:**
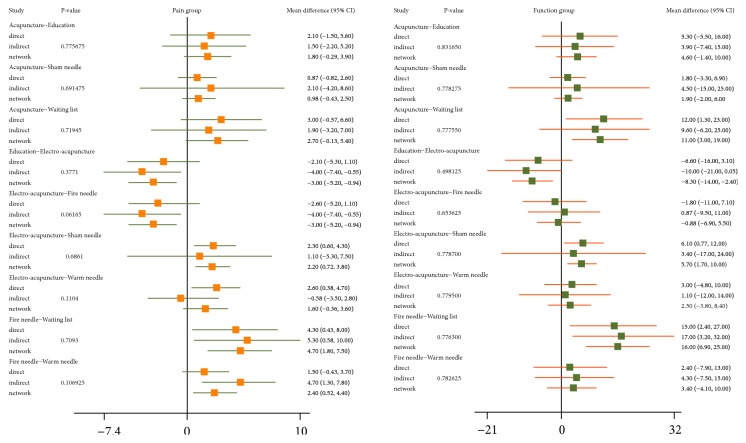
Node-splitting plots for assessing consistency with respect to the decline in (WOMAC) pain (left) and (WOMAC) physical function (right) scores.

**Figure 10:**
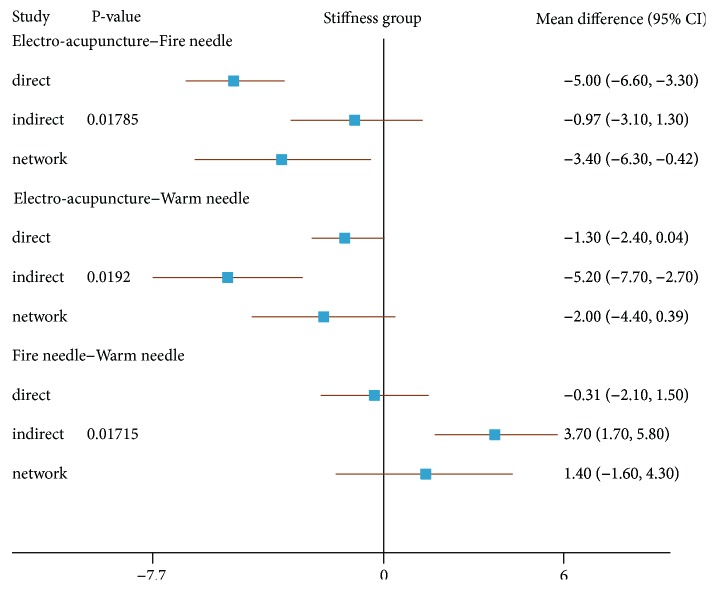
The node-splitting plot for assessing consistency with respect to the decline in (WOMAC) stiffness scores.

In order to report the inconsistency of the results, we set a node-splitting analysis and Bayesian* P* value to estimate direct and indirect evidences (Figure 9). If the* P* value in the forest plot was less than 0.05, it indicated that there was inconsistency between the direct comparison and the indirect comparison, and, on the other hand, the clinical evidence showing the final result in NMA was not credible. The node-splitting results indicated that there was no significant evidence of inconsistency between direct and indirect comparison (*P* value>0.05) in the majority of our results for pain and physical function. Hence, the consistency model's conclusion about pain and physical function was credible to this network analysis. However, it unfortunately showed inconsistency for stiffness between fire needle and electro-acupuncture (P = 0.01785), warm needle and electro-acupuncture (P = 0.0192), and fire needle and electro-acupuncture (P = 0.01715) (Figure 10). It suggested that more randomized controlled trials were needed to verify the effects of different acupuncture on improving stiffness.

### 3.7. Cumulative Contribution for the KOA Network

**Figure 11:**
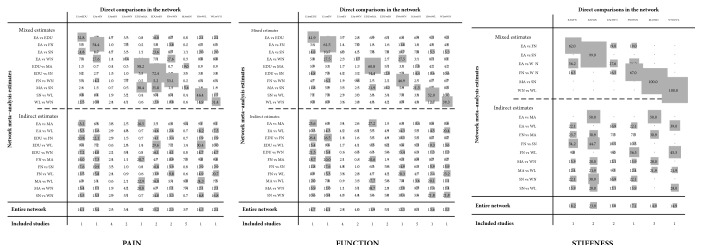
Cumulative contribution plots for the KOA network by all interventions.

Under the NMA calculation, each direct comparison contributed differently to the estimation of the network summary effects [35]. So it was necessary to evaluate the most influential comparisons for the NMA. Especially if more high-risk biased and most contributing studies were included in the NMA, the final results might be clinically untrustworthy. In cumulative contribution plots, the columns stood for all existing direct comparisons, and the rows stood for all potential pairwise comparisons. We could identify the contribution by matrices and values on it. For instance, for a contribution graph in pain group, there were ten direct comparisons (EA-EDU, EA-FN, EA-SN, EA-WN, EDU-MA, EDU-SN, FN-WN, MA-SN, SN-WL, and WL-WN). And ten direct comparisons above generated the EA-MA comparison estimate of the NMA indirectly with contributions 23.3%, 6.6%, 3.6% 2.5%, 26.5%, 3.3%, 6.6%, 9.4%, 9.1%, and 9.1%. Accordingly, when EA-MA was indirectly compared, the direct comparisons EA-EDU (23.3%) and EDU-MA (26.5%) served as the most influential ones for it. Moreover, in the entire network, the overall contribution of EDU-SN to network estimation was 15.2%, which served as the most informative and contributing direct evidence. And we could get these contribution plots by means of Stata14.0 (Figure 11).

### 3.8. Publication Bias Plot, Sensitivity Analysis, and Egger's Test

**Figure 12:**
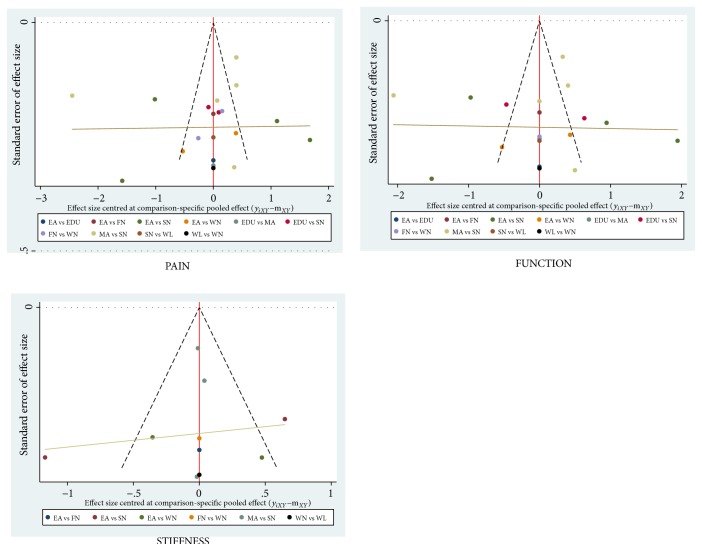
Funnel plots of all included studies referring to (WOMAC) pain, stiffness, and physical function scores.

The funnel plot of most studies included in NMA clung to the middle of the line (x =0) in vertical distribution, which indicated that there was no evidence of small-study effect (Figure 12). Although some included studies were scattered out of the interval due to the high heterogeneity, sensitivity analyses (Tables S3, S4, and S5) had confirmed the robustness of the results among all included studies, and Egger's test (Tables S1, S2, and S3) showed there was no small-study effects and bias with respect to pain, stiffness, and function. These analyses proved that the study results were less likely to be biased and unreliable. However, due to the clinical heterogeneity of the funnel plots, more high-quality researches are needed in the future.

## 4. Discussion

Osteoarthritis of knee is a chronic cataplasia disease followed with progressive joint pain, stiffness, and physical motor dysfunction. In this systematic review, we assessed different acupuncture methods for the relief of pain, stiffness, and the improvement of physical function on account of KOA. In addition to stiffness assessment of insufficient evidence, results of our NMA indicated that all acupuncture methods to reduce joint pain and restore physical function were better than other no intervention and education controls, especially that fire needle and electro-acupuncture were considered as the most efficient in KOA. However, there was no significant difference in stiffness calculations, which meant more RCTs were needed to prove it.

In recent years, studies had found that electro-acupuncture could effectively interfere with inflammatory pain and neuropathic pain. Even if electro-acupuncture was still controversial on analgesic effect, but some animal experiments [37–39] had confirmed that electro-acupuncture could relieve neuropathic pain via upregulation of glutamate transporters in the spinal cord of rats. In addition, EA triggered the release of endogenous opioids and adenosine to alleviate inflammatory pain in mice. Among them, the new viewpoint mentioned that the mechanism of electro-acupuncture analgesia could be related to the regulation of TRPV1 and p-TRPV1. But an analgesic effect of the EA to the regulating TRPV1 and p-TRPV1 remained to be further explored. Moreover, by adjusting the frequency of applied EA, we guessed that EA treatment could facilitate the release of particular neuropeptides from the central nervous system, subsequently activating self-healing mechanisms, which could contribute to functional recovery. What is more, Huang's study [40] showed that both EA and massage can effectively suppress the release of synovial IL-*β* and TNF-*α* in KOA of rabbits, which may contribute to the effects of acupuncture in the treatment of osteoarthritis. Thus, electro-acupuncture based on the role of the current method could stimulate the organization. Meanwhile the manipulation of the needle was to use a mechanical movement such as lifting the plug to stimulate the acupuncture tissue, and of which the acupuncture point was more effective.

In terms of the relief of pain, the effect of the fire needle was the best in the NMA. Law D's study [41] updated current available evidence from a recent randomized controlled trial which used high heat points to treat musculoskeletal disorders. These researches of 2/3 reported the effects of laser acupuncture on pain and functional results were positive. Like the principle of laser acupuncture on the human body, fire needle also worked by focusing energy on one part of the human body. High-energy stimuli [18] could well expand blood vessels and improve circulation, blood supply, and metabolism; especially within a narrow range around the fire needle body, the lesions were burnt to carbonization, thereby quickly ameliorating local tissue edema, hyperemia, exudation, adhesions, calcification, and contracture, in order to promote the absorption of inflammatory exudate. More opinions [42] indicated that the mechanism of fire needle of KOA might be related to the regulation of IL-1 signal transduction pathway IL-1*β* and IL-1R*α* levels and IL-1R*α*/IL-1*β* ratio, so as to adjust the articular cartilage and decomposition of the role of balance. Recent studies [43, 44] reported that owing to the active treatment of acupuncture through different experimental heat pain stimulation, the expectation of pain relief can be effectively transferred to the treatment of chronic osteoarthritis pain, which might explain the effects of the fire needle and this was a new theory based on placebo analgesia between boosted expectation and pain modulation system. However, the existing research to the effects of fire needle was still insufficient, so further exploration was needed.

In the matter of easing stiffness symptom and promoting functional recovery, warm needle and fire needle were based on acupuncture to stimulate the body with warm stimuli to better improve KOA in traditional pairwise meta-analysis. Compared to simply using acupuncture methods, these two methods could play a role of warming meridians. However, the stimuli of fire needle against the human body were short-lived and powerful. The author believed that fire needle could cause the meridians of the body to be opened by the heat of the outside world in a short period of time and prolonged warmth stimulation might cause the defence mechanism of evil and let evil entangle the body. Therefore, more clinical trials might be needed to prove the efficacy of warm needle in the long-term improvement of physical function than fire needle. Comparison between sham needle and real manual acupuncture has no statistical significance, which meant sham needle could still have an effect. At the same time, according to the ranking results, the effect of ordinary acupuncture was better than that of a warm needle in pain relief. It might be that the patient does not tolerate long-term warm stimulation and has a resistance effect. In respect of stiffness, warm needle embodied the advantage. Through the continuous warm effects and acupoint stimulation, it could relieve the soft tissue spasm of muscle, joint capsule and fascia, and improve the coordination mode of the active and antagonistic muscle activities with quadriceps femoris, hamstring and popliteus tendon [13]. But in the inconsistency of stiffness result, we needed more RCTs to proof it. Apart from statistical significance, we believed that the clinical significance of warm needle would be more worthy of attention than manual acupuncture.

From where we stood, fire needle with transient high-calorie shocks and electro-acupuncture with persistent electrical stimulation might cause some pain metastases to knee osteoarthritis, and warm needle with continuous warming cause the surrounding tissue to gradually relax, which could improve the range of motion in patients. Among the three indicators in the WOMAC score, a study [45] showed that the highest reliability of the physical function assessment was up to 92%, the pain assessment was 74% in the second place, and the reliability of the stiffness assessment was the lowest, only 58%, which were also similar to the results of our study based on inconsistency test in NMA. And another study [46] reported that its pain scale was too highly related to physical function. It made us fail to take joint-specific pain measures in direct validity. So the pain and function, simultaneously, and the stiffness assessment needed more outcome methods to measure in further research.

## 5. Limitations

### 5.1. Limited Methodological Quality of Included Studies

The methodological quality evaluation was low. Some Chinese RCTs did not describe blind method and follow-up time. Some English RCTs blind methods were not clear, which were prone to subjective bias. Individual study samples were less abundant. Although acupuncture was difficult to do blindly, we could also design a single blind between researchers, acupuncturists, and patients to improve the quality of evidence.

### 5.2. Limited Measurements

Long-term efficacy had not yet been achieved in this NMA. Meanwhile, most of the articles failed to illustrate the adverse reactions and compliance; for example, whether the long-term effect of the fire needle and warm needle might cause skin damage to the joints, whether the acceptance would gradually decline, or whether the electro-acupuncture would give patients nerve fatigue in the long-term effect. Also because the NMA method could achieve diversified and multidimensional analysis in multiple outcome indicators, we only focused more on the relationship between the effects of different acupuncture methods and pain, stiffness, and physical function in osteoarthritis of the knee on basis of WOMAC score in the article. But the NMA results reminded us that we could also pay more attention to other knee scoring systems, such as the Lysholm score, the American knee society score (AKS), and the knee injury and osteoarthritis score (KOOS) in subsequent research. And for acupuncture and moxibustion treatment, many studies were short of objective outcome indicators, such as the measurement of TNF-*α*, IL-1*β* level in synovial fluid of the knees, or the results of bone level in thermal tomography system (TTM), ultrasonography or MRI. We needed more uniform and standardized therapeutic criteria to prove up the relationship between acupuncture and osteoarthritis and its mechanism.

### 5.3. Limited Experimental Design in Acupuncture

Acupuncture had a certain effect along with heat pain stimulation, but lacked accuracy. Like fire needle and warm needle, they did not have a precise temperature change setting and the depth of acupuncture in comparable baseline. Moreover considering electro-acupuncture as another means of curative effect, many studies did not regulate its electrical stimulation frequency, duration, and depth. All in all, the risk of expected bias could always be magnified by irregular operations or the control design by blinding the control participants, different manipulations of doctors, or degree on content of compliance in patients, etc. Inconsistent follow-up time, treatment duration, and demographic characteristics could also result in heterogeneity of outcome.

## 6. Conclusions

As a result, this NMA suggests that fire needle and electro-acupuncture may be potential acupuncture methods to relieve the pain of patients with KOA and largely improve physical function of daily life. Regarding the comparisons of different acupuncture methods in improving stiffness sense, more evidence is needed. This NMA demonstrates that acupuncture with accurate and individual heat pain and electrical stimulation can have a significant effect. This result may represent a possible trend of the acupuncture research in the future to motivate the potential of the mechanism of the body recovery in osteoarthritis of the knee or other diseases. However, most studies have reported only short-term efficacy, so it is still necessary to further validate multiple long-term and high-quality randomized controlled studies based on large sample data, within standardize unified operations.

## Figures and Tables

**Table 1:** Characteristics of the included studies.

Author, Year	Location	Sample size / Gender		Mean Age		Interventions				Follow up
		T(M/F)	C(M/F)	T	C	T	Sessions/ Duration (n/ws)	C	Measurement Time Points (ws)	
Berman 2004 [26]	USA	190(70/120)	189(62/127)	65.2±8.4	65.1±8.8	EA	23/26	EDU	4/8/14/26	26ws
			191(73/118)	66.2±8.7				SN		
Berman 1999 [27]	USA	36	37	65.7±7.95	65.5±9.13	MA	16/8	WL	4/8/12	12ws
Zhou 2017[18]	China	54(25/29)	56(23/33)	65±6	63±6	FN	12/4	EA	4/8	NA
Hinman 2014[28]	Australia	70(38/32)	71(31/40)	64.3±8.6	62.7±8.7	MA	8,12/12	SN	12/26	12ms
Lu 2014[17]	China	30(16/14)	30(18/12)	58.93±9.26	59.10±7.85	EA	24/8	WN	8	NA
Manheimer 2006 [29]	USA	190(70/120)	189(62/127)	65.2±8.4	65.1±8.8	MA	16,12/8,12	EDU	4/8/14/26	26ws
			191(73/118)	66.2±8.7				SN		
Sangdee 2002 [9]	Thailand	48(10/38)	49(10/39)	65.10±3.4	61.84±8.95	EA	12/4	SN	4	4ws
Zhang 2013 [16]	China	33(13/20)	34(14/20)	57±8	58±9	FN	12/4	WN	4	NA
Scharf 2006 [10]	Germany	326(106/220)	365(110/255)	62.8±9.9	63.0±10.1	MA	10/6	SN	13/26	26ws
Takeda 1994 [11]	Canada	20(10/10)	20(10/10)	63.0±8.78	60.2±9.75	MA	9/3	SN	3/7	4ws
Vas 2004 [12]	Spain	48(11/37)	49(5/44)	65.7±11.0	68.4±9.1	EA	12/12	SN	13	12ws
Fan 2016 [30]	China	54(21/33)	54(24/30)	58 ± 6.2	56 ± 8.4	FN	8/4	WN	1/4	NA
Wang 2017 [13]	China	25	21	61±6	58±7	WN	12/3	WL	3	6ds
Chen 2013 [14]	USA	104(51/53)	109(52/57)	60.5±11.1	60.4±11.7	EA	12/⩽12	SN	12/26	26ws
Jubb 2008 [31]	USA	34	35	64.1±1.6	66.1±1.9	EA	10/5	SN	9	9ms
Gao 2012 [15]	China	34(13/21)	35(15/20)	57.7±8.7	58.6±8.9	EA	24/8	WN	4/8	NA

M: male; F: female; T: treatment group; C: control group; NA: not available; EA: electro-acupuncture; SN: sham needle; MA: manual acupuncture; WN: warm needle; FN: fire needle; WL: waiting list; EDU: education; n: number; ws: weeks; ms: months; ds: days.

**Table 2:** Results (MD, with 95% CI) of the pairwise meta-analysis for pain and function scores.

	Sham needle vs Education	Acupuncture vs Sham needle	Electro-acupuncture vs Sham needle	Electro-acupuncture vs Warm needle	Acupuncture vs Waiting list	Acupuncture vs Education	Electro-acupuncture vs Education	Fire needle vs Electro-acupuncture	Fire needle vs Warm needle	Warm needle vs Waiting list
Pain	***-1.14 [-1.20, -1.08]***	***-0.68 [-1.06, -0.31]***	***-2.25 [-3.42, -1.08]***	***-2.58 [-4.77, -0.39]***	***-3.01 [-4.71, -1.31]***	***-2.09 [-2.17, -2.01]***	***-2.09 [-2.17, -2.01]***	***-2.57 [-3.67, -1.47]***	-1.70 [-4.25, 0.85]	***-4.26 [-6.50, -2.02]***

Function	***-3.78 [-3.95, -3.61]***	-1.74 [-3.82, 0.33]	***-5.92 [-9.43, -2.41]***	-2.36 [-12.27, 7.55]	***-11.98 [-18.01, -5.95]***	***-6.56 [-6.83, -6.29]***	***-6.56 [-6.80, -6.32]***	***-1.80 [-3.11, -0.49]***	-2.40 [-7.78, 2.98]	***-14.70 [-23.86, -5.54]***

*∗*Boldface and italic meant significance. (Note: if MD<0, it meant that the therapeutic group was better than the control group.)

**Table 3:** Results (MD, with 95% CI) of the network meta-analysis for pain (on the bottom left) and function (on the top right) scores.

**Acupuncture**	4.50(-1.00, 10.00)	-3.70(-9.30, 1.50)	-4.60(-12.00, 2.80)	1.90(-1.90, 5.90)	***11.00(3.00, 19.00)***	-1.20(-9.40, 6.30)
-1.80(-4.00, 0.29)	**Education**	***-8.30(-14.00, -2.60)***	***-9.20(-17.00, -0.98)***	-2.70(-7.70, 2.70)	6.6(-3.00, 16.00)	-5.90(-14.00, 2.10)
1.20(-0.75, 3.20)	***3.00(0.92, 5.20)***	**Electro-acupuncture**	-0.88 (-6.90, 5.40)	***5.70(1.70, 10.00)***	***15.00(6.30, 24.00)***	2.50 (-3.70, 8.30)
2.00(-0.63, 4.70)	***3.90(1.00, 6.80)***	0.84(-1.30, 2.90)	**Fire needle**	6.60(-0.35, 14.00)	***16.00(7.00, 25.00)***	3.40 (-3.90, 10.00)
-0.98 (-2.50, 0.43)	0.85 (-1.10, 2.70)	***-2.20(-3.80, -0.72)***	***-3.00(-5.50,-0.59)***	**Sham needle**	***9.30(0.61, 18.00)***	-3.20(-11.00, 3.60)
-2.70(-5.40, 0.14)	-0.85 (-4.10, 2.50)	***-3.90(-6.80, -0.89)***	***-4.10(-6.70, -1.50)***	***-4.70(-7.60, -1.80)***	**Waiting list**	***-13.00(-23.00, -2.80)***
-0.40 (-3.20, 2.30)	1.40 (-1.50, 4.30)	-1.60(-3.70, 0.37)	***-2.40(-4.40, -0.51)***	0.59(-1.90, 3.10)	2.30(-1.00, 5.40)	**Warm needle**

*∗*Boldface and italic meant significance. (Note: if MD<0, it meant that the treatment in columns was more effective than that of the rows in NMA.)

**Table 4:** Results (MD, with 95% CI) of the network meta-analysis for stiffness (on the bottom left) and pairwise meta-analysis for stiffness (on the top right) scores.

**Acupuncture**	-	-	0.15 [-0.17, 0.46]	-	-
1.02 (-2.50, 4.52)	**Electro-acupuncture**	***-4.95 [-5.63, -4.27]***	***1.09 [0.23, 1.96]***	-	***-1.24 [-2.06, -0.42]***
4.43 (-0.13, 9.02)	***3.41(0.34, 6.34)***	**Fire needle**	-	-	-0.30 [-1.32, 0.72]
-0.20 (-2.52, 2.21)	-1.20 (-3.84, 1.54)	***-4.60(-8.62, -0.65)***	**Sham needle**	-	-
1.20 (-4.60, 7.03)	0.19 (-4.51, 4.82)	-3.19 (-8.06, 1.68)	1.39 (-3.99, 6.70)	**Waiting list**	***-1.80 [-2.90, -0.70]***
3.00 (-1.23, 7.25)	1.96 (-0.49, 4.44)	-1.43 (-4.25, 1.62)	3.18 (-0.35, 6.79)	1.77 (-1.99, 5.73)	**Warm needle**

*∗*Boldface and italic meant significance. (Note: if MD<0, it meant that the treatment in columns was more effective than that of the rows.)

**Table 5:** Rank probabilities of all treatments with SUCRA values.

**Treatment**	**Pain**	**Rank**	**Function**	**Rank**	**Stiffness**	**Rank**
Acupuncture	0.39	3	0.44	4	0.63	4
Education	0.83	6	0.80	6	-	-
Electro-acupuncture	0.16	2	0.15	2	0.47	3
Fire needle	0.04	1	0.11	1	0.04	1
Sham needle	0.64	5	0.63	5	0.69	5
Waiting list	0.92	7	0.98	7	0.47	3
Warm needle	0.52	4	0.38	3	0.18	2

*∗*The larger the SUCRA value in this NMA, the worse the rank of the treatment.

## Supplementary Materials

Supplementary Materials Tables S1, S2, and S3: subgroup analysis and Egger's test. Tables S4, S5, and S6: sensitivity analysis.
