# Corrigendum to “Efficacy Comparison of Five Different Acupuncture Methods on Pain, Stiffness, and Function in Osteoarthritis of the Knee: A Network Meta-Analysis”

**DOI:** 10.1155/2019/3713197

**Published:** 2019-11-20

**Authors:** Shaowei Li, Pingjin Xie, Zhenghui Liang, Weihan Huang, Zhanhui Huang, Jinming Ou, Zhiyong Lin, Shengting Chai

**Affiliations:** ^1^Guangzhou University of Chinese Medicine, Guangzhou 510405, Guangdong, China; ^2^Department of Orthopaedics, The Affiliated Orthopaedics and Trauma Hospital of Guangzhou University of Chinese Medicine, Guangzhou 510240, Guangdong, China

In the article titled “Efficacy Comparison of Five Different Acupuncture Methods on Pain, Stiffness, and Function in Osteoarthritis of the Knee: A Network Meta-Analysis” [1], there were missing inclusion criteria in the “2. Materials and Methods” of Page 2, where “Interventions were compared between common manual acupuncture, electro-acupuncture, fire needle, warm needle, placebo, sham needle, or education” should be corrected to “Interventions included common manual acupuncture, electro-acupuncture, fire needle, or warm needle. Comparators were any of the above described interventions compared with each other, or placebo, waiting list control (including no intervention), sham needle, or education. There was no language restriction in search strategy.” Additionally, there were errors in the typesetting and description in Table 1. The corrected table is shown below.

## Supplementary Materials

Supplementary Materials The relevant corrected values which were re-estimated in the study.

## Figures and Tables

**Table 1:** Characteristics of the included studies.

Author, Year	Location	Sample size/Gender		Mean Age		Interventions				Follow up
		T(M/F)	C(M/F)	T	C	T	Sessions/Duration	C	Measurement Time Points	
							(n/ws)			
									(ws)	
Berman 2004[26]	USA	190(70/120)	189(62/127)	65.2±8.4	65.1±8.8	EA	23/26	EDU	4/8/14/26	-
			191(73/118)		66.2±8.7			SN		
Berman 1999[27]	USA	36(18/18)	37(26/11)	65.7±7.95	65.5±9.13	EA	16/8	WL	4/8/12	4ws
Zhou 2017[18]	China	56(23/33)	54(25/29)	65±6	63±6	FN	12/4	EA	4/8	4ws
Hinman 2014[28]	Australia	70(38/32)	71(31/40)	64.3±8.6	62.7 ± 8.7	MA	8,12/12	WL	12/50	12ms
Lu 2014[17]	China	30(16/14)	30(18/12)	58.93±9.26	59.10±7.85	EA	24/8	WN	8	NA
Manheimer 2006[29]	USA	190(70/120)	189(62/127)	65.2±8.4	65.1±8.8	EA	23/26	EDU	4/8/14/26	-
			191(73/118)		66.2±8.7					
								SN		
Sangdee 2002[9]	Thailand	48(10/38)	47(12/35)	65.10±3.4	61.84±8.95	EA	12/4	SN	4	NA
Zhang 2013[16]	China	33(13/20)	34(14/20)	57±8	58±9	FN	12/4	WN	4	NA
Scharf 2006[10]	Germany	326(106/220)	365(110/255)	62.8±9.9	63.0±10.1	MA	10/6	SN	13/26	20ws
Takeda 1994[11]	Canada	20(10/10)	20(10/10)	63.0±8.78	60.2±9.75	MA	9/3	SN	3/7	4ws
Vas 2004[12]	Spain	48(11/37)	49(5/44)	65.7±11.0	68.4±9.1	EA	12/12	SN	13	7ds
Fan 2016[30]	China	54(21/33)	54(24/30)	58 ± 6.2	56 ± 8.4	FN	8/4	WN	1/4	-
Wang 2017[13]	China	25(8/17)	21(2/19)	61±6	58±7	WN	12/3	WL	3	NA
Chen 2013[14]	USA	104(51/53)	109(52/57)	60.5±11.1	60.4±11.7	MA	12/⩽12	SN	12/26	14ws
Jubb 2008[31]	UK	34(29/5)	34(26/8)	64.1±1.6	66.1±1.9	EA	10/5	SN	5/9	4ws
Gao 2012[15]	China	34(13/21)	35(15/20)	57.7±8.7	58.6±8.9	EA	24/8	WN	4/8	NA

M: male; F: female; T: treatment group; C: control group; NA: not available; EA: electroacupuncture; SN: sham needle; MA: manual acupuncture; WN: warm needle; FN: fire needle; WL: waiting list; EDU: education; n: number; ws: weeks; ms: months; ds: days.
