# Supplementary material for: Efficacy Comparison of Five Different Acupuncture Methods on Pain, Stiffness, and Function in Osteoarthritis of the Knee: A Network Meta-Analysis
Source: Evid Based Complement Alternat Med. 2018 Nov 1;2018:1638904. doi: 10.1155/2018/1638904 (PMC6236871; doi:10.1155/2018/1638904)
Supplement: Supplementary Materials — Tables S1, S2, and S3: subgroup analysis and Egger's test. Tables S4, S5, and S6: sensitivity analysis. [file 1638904.f1.doc]

## Supplementary Materials

**Table S1**: Subgroup Analysis and Egger’s Test of the included studies of pain.

| Pain | Treatment group | Control group | t | ***P*-value** |
| --- | --- | --- | --- | --- |
|  | Acupuncture& Electro-acupuncture | Sham needle | -1.40 | 0.204 |
|  |  | -1.16, [-1.51, -0.82] |  |  |
|  | All Acupuncture methods | Education and No intervention | -0.380 | 0.721 |
|  |  | -2.09, [-2.15, -2.03] |  |  |
|  | **(H0: no small-study effects)** |  |  |  |

**Table S2**: Subgroup Analysis and Egger’s Test of the included studies of physical function.

| Function | Treatment group | Control group | t | ***P*-value** |
| --- | --- | --- | --- | --- |
|  | Acupuncture& Electro-acupuncture | Sham needle | -1.26 | 0.248 |
|  |  | -3.34, [-4.68,-1.99] |  |  |
|  | All Acupuncture methods | Education and No intervention | -0.22 | 0.836 |
|  |  | -6.60, [-6.97, -6.22] |  |  |
|  | **(H0: no small-study effects)** |  | | |

**Table S3**: Subgroup Analysis and Egger’s Test of the included studies of stiffness.

| Stiffness | Treatment group | Control group | t | ***P*-value** |
| --- | --- | --- | --- | --- |
|  | Acupuncture& Electro-acupuncture & Warm needle | Sham needle & No intervention | -1.77 | 0.151 |
|  |  | -0.687, [-1.264, -0.110] |  |  |
|  | **(H0: no small-study effects)** |  | | |

**Table S4**: Sensitivity Analysis of the included studies of pain.

|  | **MD 95%CI** | ***P*-value** | **tau^2^** | ***I^2^*** |
| --- | --- | --- | --- | --- |
| **Sham needle VS Education** |  |  |  |  |
| Omitting Berman 2004 | -1.14 [-1.22, -1.06] | < 0.001 | 0.00 | - |
| Omitting Manheimer 2006 | -1.14 [-1.22, -1.06] | < 0.001 | 0.00 | - |
| **Pooled estimate** | -1.14 [-1.20, -1.08] | < 0.001 | 0.00 | 0.00 |
| **Acupuncture VS Sham needle** |  |  |  |  |
| Omitting Chen 2013 | -0.81 [-1.38, -0.25] | 0.005 | 0.18 | 0.78 |
| Omitting Hinman 2014 | -0.60 [-0.99, -0.21] | 0.002 | 0.11 | 0.97 |
| Omitting Manheimer 2006 | -0.48 [-0.77, -0.19] | 0.17 | 0.03 | 0.40 |
| Omitting Scharf 2006 | -0.82 [-1.26, -0.38] | 0.0003 | 0.11 | 0.97 |
| Omitting Takeda 1994 | -0.68 [-1.06, -0.30] | 0.0004 | 0.11 | 0.97 |
| **Pooled estimate** | -0.68 [-1.06, -0.31] | 0.001 | 0.11 | 0.96 |
| **Electro-acupuncture VS Sham needle** |  |  |  |  |
| Omitting Berman 2004 | -2.94 [-4.55, -1.33] | 0.0003 | 1.42 | 0.71 |
| Omitting Jubb 2008 | -2.33 [-3.61, -1.04] | 0.0004 | 1.07 | 0.98 |
| Omitting Sangdee 2002 | -2.47 [-4.94, -0.01] | 0.05 | 4.05 | 0.88 |
| Omitting Vas 2004 | -1.68 [-2.90, -0.45] | 0.007 | 0.94 | 0.98 |
| **Pooled estimate** | -2.25 [-3.42, -1.08] | 0.0002 | 1..05 | 0.97 |
| **Electro-acupuncture VS Warm needle** |  |  |  |  |
| Omitting Gao 2012 | -3.57 [-5.06, -2.08] | < 0.001 | 0.00 | - |
| Omitting Lu 2014 | -1.32 [-3.45, 0.81] | 0.02 | 0.00 | - |
| **Pooled estimate** | -2.58 [-4.77, -0.39] | 0.02 | 1.05 | 0.65 |
| **Fire needle VS Warm needle** |  |  |  |  |
| Omitting Fan 2016 | -3.20 [-5.26, -1.14] | 0.002 | 0.00 | - |
| Omitting Zhang 2013 | -0.57 [-1.22, 0.08] | 0.08 | 0.00 | - |
| **Pooled estimate** | -1.70 [-4.25, 0.85] | 0.19 | 2.85 | 0.82 |

MD: mean difference, CI: confidence interval.

**Table S5**: Sensitivity Analysis of the included studies of physical function.

|  | **MD 95%CI** | ***P*-value** | **tau^2^** | ***I^2^*** |
| --- | --- | --- | --- | --- |
| **Sham needle VS Education** |  |  |  |  |
| Omitting Berman 2004 | -3.78 [-4.02, -3.54] | < 0.001 | 0.00 | - |
| Omitting Manheimer 2006 | -3.78 [-4.02, -3.54] | < 0.001 | 0.00 | - |
| **Pooled estimate** | -3.78 [-3.95, -3.61] | < 0.001 | 0.00 | 0.00 |
| **Acupuncture VS Sham needle** |  |  |  |  |
| Omitting Chen 2013 | -2.13 [-4.43, 0.18] | 0.07 | 3.30 | 0.98 |
| Omitting Hinman 2014 | -1.16 [-3.38, 1.05] | 0.30 | 3.24 | 0.98 |
| Omitting Manheimer 2006 | -1.10 [-3.39, 1.19] | 0.35 | 2.41 | 0.47 |
| Omitting Scharf 2006 | -2.56 [-4.37, -0.75] | 0.006 | 1.28 | 0.32 |
| Omitting Takeda 1994 | -1.78 [-3.86, 0.31] | 0.09 | 3.28 | 0.98 |
| **Pooled estimate** | -1.74 [-3.82, 0.33] | 0.10 | 3.26 | 0.97 |
| **Electro-acupuncture VS Sham needle** |  |  |  |  |
| Omitting Berman 2004 | -7.75 [-14.62, -0.88] | 0.03 | 30.18 | 0.84 |
| Omitting Jubb 2008 | -7.02 [-10.87, -3.17] | 0.0004 | 9.42 | 0.98 |
| Omitting Sangdee 2002 | -6.22 [-14.31, 1.88] | 0.13 | 44.27 | 0.89 |
| Omitting Vas 2004 | -3.98 [-7.56, -0.39] | 0.03 | 8.01 | 0.98 |
| **Pooled estimate** | -5.92 [-9.43, -2.41] | 0.0009 | 9.26 | 0.97 |
| **Electro-acupuncture VS Warm needle** |  |  |  |  |
| Omitting Gao 2012 | -7.00 [-11.66, -2.34] | 0.003 | 0.00 | - |
| Omitting Lu 2014 | 3.15 [-4.31, 10.61] | 0.41 | 0.00 | - |
| **Pooled estimate** | -2.36 [-12.27, 7.55] | 0.64 | 21.26 | 0.80 |

MD: mean difference, CI: confidence interval.

**Table S6**: Sensitivity Analysis of the included studies of stiffness.

|  | **MD 95%CI** | ***P*-value** | **tau^2^** | ***I^2^*** |
| --- | --- | --- | --- | --- |
| **Acupuncture VS Sham needle** |  |  |  |  |
| Omitting Chen 2013 | 0.20 [-0.19, 0.60] | 0.32 | 0.00 | 0.00 |
| Omitting Scharf 2006 | 0.06 [-0.45, 0.57] | 0.82 | 0.00 | 0.00 |
| Omitting Takeda 1994 | 0.14 [-0.17, 0.46]] | 0.37 | 0.00 | 0.00 |
| **Pooled estimate** | 0.15 [-0.17, 0.46] | 0.36 | 0.00 | 0.00 |
| **Electro-acupuncture VS Sham needle** |  |  |  |  |
| Omitting Sangdee 2002 | 1.70 [0.69, 2.71] | 0.001 | 0.00 | - |
| Omitting Vas 2004 | 0.77 [0.64, 0.90] | < 0.001 | 0.00 | - |
| **Pooled estimate** | 1.09 [0.23, 1.96] | 0.01 | 0.18 | 0.69 |
| **Warm needle VS Electro-acupuncture** |  |  |  |  |
| Omitting Gao 2012 | -0.74 [-1.72, 0.24] | 0.14 | 0.00 | - |
| Omitting Lu 2014 | -1.59 [-2.27, -0.91] | < 0.001 | 0.00 | - |
| **Pooled estimate** | -1.24 [-2.06, -0.42] | 0.02 | 1.05 | 0.49 |

MD: mean difference, CI: confidence interval.
