# Supplementary material for: Corrigendum to “Efficacy Comparison of Five Different Acupuncture Methods on Pain, Stiffness, and Function in Osteoarthritis of the Knee: A Network Meta-Analysis”
Source: Evid Based Complement Alternat Med. 2019 Nov 20;2019:3713197. doi: 10.1155/2019/3713197 (PMC6935462; doi:10.1155/2019/3713197)
Supplement: Supplementary Materials — The relevant corrected values which were re-estimated in the study. [file 3713197.f1.pdf]

Results (MD, with 95% CI) of the network meta-analysis for pain scores.

Acupuncture	0.97 (-1.87, 3.72)	-1.70 (-3.98, 0.53)	-2.96 (-6.09, 0.16)	0.35 (-1.66, 2.34)	2.16 (-0.28, 4.74)	-0.62 (-3.38, 2.24)
-0.97 (-3.72, 1.87)	Education	***-2.68 (-4.78, -0.65)***	***-3.94 (-6.97, -0.85)***	-0.64 (-2.62, 1.50)	1.21 (-1.72, 4.23)	-1.60 (-4.40, 1.22)
1.70 (-0.53, 3.98)	***2.68 (0.65, 4.78)***	Electro-acupuncture	-1.26 (-3.50, 1.06)	***2.04 (0.67, 3.55)***	***3.88 (1.64, 6.28)***	1.09 (-0.78, 3.07)
2.96 (-0.16, 6.09)	***3.94 (0.85, 6.97)***	1.26 (-1.06, 3.50)	Fire needle	***3.31 (0.68, 5.99)***	***5.14 (2.20, 8.24)***	***2.34 (0.35, 4.45)***
-0.35 (-2.34, 1.66)	0.64 (-1.50, 2.62)	***-2.04 (-3.55, -0.67)***	***-3.31 (-5.99, -0.68)***	Sham needle	1.83 (-0.57, 4.30)	-0.95 (-3.31, 1.36)
-2.16 (-4.74, 0.28)	-1.21 (-4.23,1.72)	***-3.88 (-6.28, -1.64)***	***-5.14 (-8.24, -2.20)***	-1.83 (-4.30, 0.57)	Waiting list	***-2.78 (-5.38, -0.28)***
0.62 (-2.24, 3.38)	1.60 (-1.22, 4.40)	-1.09 (-3.07, 0.78)	***-2.34 (-4.45, -0.35)***	0.95 (-1.36, 3.31)	***2.78 (0.28, 5.38)***	Warm needle

*Boldface and italic meant significance. (Note: if MD<0, it meant that the treatment in columns was more effective than that of the rows in NMA.)

Results (MD, with 95% CI) of the network meta-analysis for function scores.

Acupuncture	1.53 (-6.41, 8.71)	-5.65 (-12.29, 0.04)	-7.10 (-16.85, 1.57)	-0.41 (-5.92, 4.87)	7.01 (-0.23, 14.16)	-4.14 (-12.90, 3.28)
-1.53 (-8.71, 6.41)	Education	***-7.20 (-12.78, -1.95)***	***-8.64 (-17.58, -0.16)***	-1.98 (-7.38, 3.82)	5.48 (-2.72, 14.27)	-5.66 (-14.06, 1.78)
5.65 (-0.04, 12.29)	***7.20 (1.95, 12.78)***	Electro-acupuncture	-1.44 (-8.36, 5.23)	***5.23 (1.67, 9.45)***	***12.74 (6.12, 19.78)***	1.50 (-4.39, 7.02)
7.10 (-1.57, 16.85)	***8.64 (0.16, 17.58)***	1.44 (-5.23, 8.36)	Fire needle	6.67 (-0.71, 14.71)	***14.14 (5.24, 23.91)***	3.00 (-4.57, 10.05)
0.41 (-4.87, 5.92)	1.98 (-3.82, 7.38)	***-5.23 (-9.45, -1.67)***	-6.67 (-14.71, 0.71)	Sham needle	***7.51 (0.36, 14.56)***	-3.75 (-10.89, 2.55)
-7.01 (-14.16, 0.23)	-5.48 (-14.27, 2.72)	***-12.74(-19.78,-6.12)***	***-14.14 (-23.91, -5.24)***	***-7.51 (-14.56, -0.36)***	Waiting list	***-11.21 (-19.40, -3.92)***
4.14 (-3.28, 12.90)	5.66 (-1.78, 14.06)	-1.50 (-7.02, 4.39)	-3.00 (-10.05, 4.57)	3.75 (-2.55, 10.89)	***11.21 (3.92, 19.40)***	Warm needle

*Boldface and italic meant significance. (Note: if MD<0, it meant that the treatment in columns was more effective than that of the rows in NMA.)

Node-splitting plots for assessing consistency with respect to the decline in (WOMAC) pain scores.

Name	Direct Effect	Indirect Effect	Overall	P-Value
Acupuncture, Sham needle	0.48 (-1.83, 2.96)	-0.31 (-5.09, 4.50)	0.35 (-1.66, 2.34)	0.75
Acupuncture, Waiting list	1.80 (-1.88, 5.44)	2.56 (-1.36, 6.71)	2.16 (-0.28, 4.74)	0.75
Electro-acupuncture, Fire needle	-2.66 (-5.81, 0.47)	0.19 (-3.00, 3.18)	-1.26 (-3.50, 1.06)	0.18
Electro-acupuncture, Sham needle	2.01 (0.49, 3.68)	2.74 (-2.38, 7.97)	2.04 (0.67, 3.55)	0.76
Electro-acupuncture, Waiting list	3.00 (-0.68, 6.67)	4.45 (1.27, 7.76)	3.88 (1.64, 6.28)	0.53
Electro-acupuncture, Warm needle	2.65 (0.51, 4.62)	-1.15 (-3.53, 1.40)	1.09 (-0.78, 3.07)	0.03
Fire needle, Warm needle	1.53 (-0.58, 3.85)	4.47 (0.70, 7.98)	2.34 (0.35, 4.45)	0.18
Waiting list, Warm needle	-4.23 (-8.10, -0.47)	-1.64 (-5.04, 1.84)	-2.78 (-5.38, -0.28)	0.29

Node-splitting plots for assessing consistency with respect to the decline in (WOMAC) physical function scores.

Name	Direct Effect	Indirect Effect	Overall	P-Value
Acupuncture, Sham needle	-0.02 (-6.35, 6.01)	-2.73 (-16.16, 10.89)	-0.41 (-5.92, 4.87)	0.68
Acupuncture, Waiting list	5.72 (-4.19, 15.67)	8.64 (-3.29, 20.03)	7.01 (-0.23, 14.16)	0.66
Electro-acupuncture, Fire needle	-1.83 (-11.24, 7.48)	-0.32 (-13.64, 11.99)	-1.44 (-8.36, 5.23)	0.83
Electro-acupuncture, Sham needle	5.09 (1.08, 9.65)	8.14 (-6.51, 22.86)	5.23 (1.67, 9.45)	0.66
Electro-acupuncture, Waiting list	12.04 (1.18, 22.55)	13.18 (4.18, 23.19)	12.74 (6.12, 19.78)	0.87
Electro-acupuncture, Warm needle	3.13 (-4.57, 10.28)	-0.93 (-10.73, 8.77)	1.50 (-4.39, 7.02)	0.47
Fire needle, Warm needle	2.51 (-8.08, 13.24)	3.47 (-8.47, 14.82)	3.00 (-4.57, 10.05)	0.87
Waiting list, Warm needle	-14.36 (-26.78, -2.09)	-9.04 (-19.32, 0.61)	-11.21 (-19.40, -3.92)	0.48

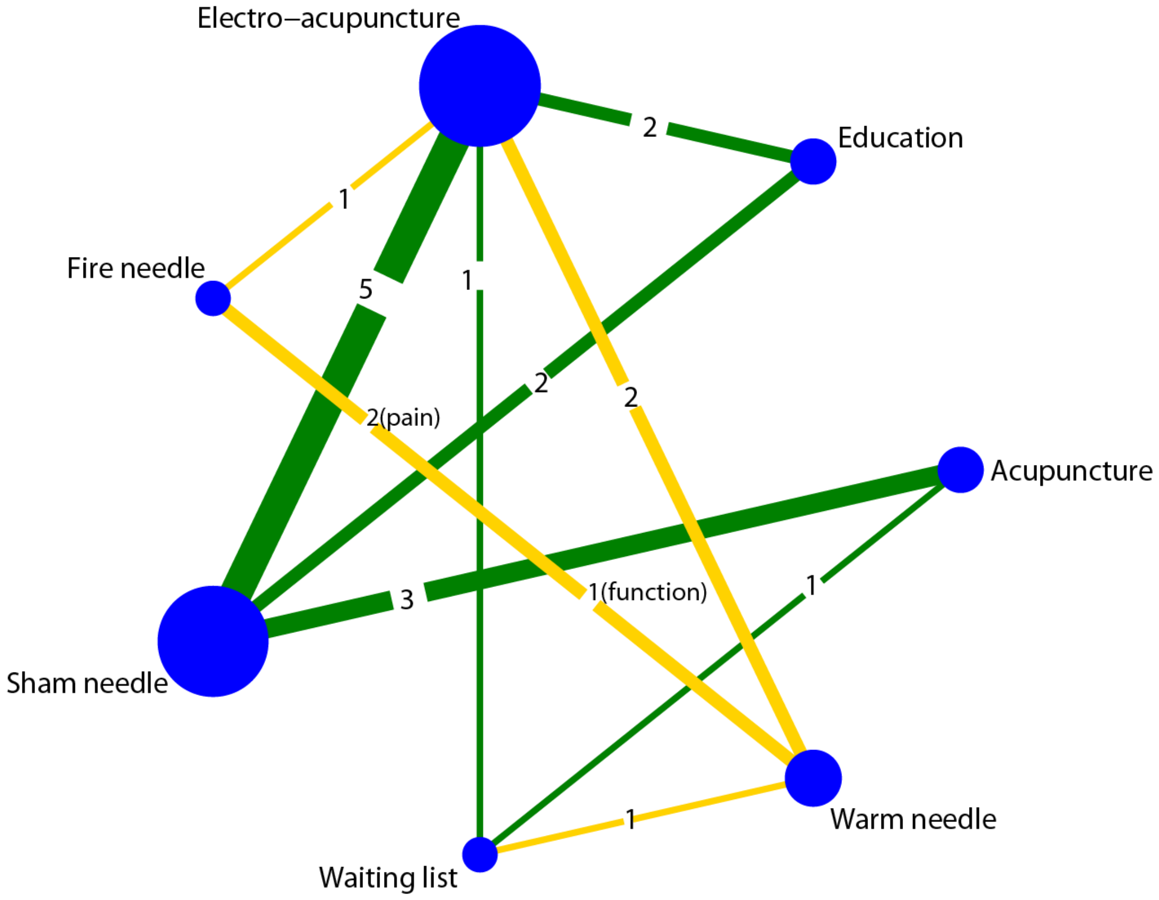

The Network comparison of acupuncture methods for KOA in pain and function.

PAIN

Direct comparisons in the network (columns); Network meta-analysis estimates (rows)

	EAvsEDU	EAvsFN	EAvsSN	EAvsWL	EAvsWN	EDUvsSN	FNvsWN	MAvsSN	MAvsWL	WLvsWN
**Mixed estimates**										
EAvsEDU	30.2	2.9	3.4	10.1	1.1	17.4	2.9	14.1	14.1	4.0
EAvsFN	3.3	48.6	0.7	5.5	6.1	3.3	15.5	3.9	3.9	9.4
EAvsSN	19.6	3.3	3.9	11.7	1.3	19.6	3.3	16.3	16.3	4.6
EAvsWL	12.4	5.9	2.5	20.7	2.3	12.4	5.9	14.9	14.9	8.2
EAvsWN	4.8	23.4	1.0	8.1	9.0	[illegible]	23.4	5.8	5.8	13.8
EDUvsSN	9.9	1.6	1.9	5.7	0.6	60.3	1.6	8.0	8.0	2.3
FNvsWN	3.4	15.9	0.7	5.6	6.3	3.4	47.2	[illegible]	4.0	9.6
MAvsSN	2.4	0.6	0.5	2.1	0.2	2.4	0.6	87.5	2.9	0.8
MAvsWL	10.6	2.6	2.1	9.1	1.0	10.6	2.6	12.7	45.3	3.6
WLvsWN	7.2	14.9	1.4	12.0	5.7	7.2	14.9	8.6	8.6	19.4
**Indirect estimates**										
EAvsMA	17.4	3.4	3.5	11.8	1.3	17.4	3.4	20.9	16.4	4.6
EDUvsFN	18.2	21.8	1.9	4.0	2.2	13.0	9.4	11.1	11.1	7.2
EDUvsMA	7.5	1.3	1.0	4.7	0.5	37.2	1.3	38.2	6.5	1.8
EDUvsWL	12.4	2.6	0.5	9.2	1.0	23.0	2.6	22.5	22.5	3.6
EDUvsWN	15.7	13.2	1.5	1.1	5.1	13.9	13.2	12.5	12.5	11.3
FNvsMA	11.9	19.2	2.4	6.6	1.7	11.9	9.5	14.3	14.4	7.8
FNvsSN	12.8	19.6	2.6	6.0	1.8	12.8	9.4	13.6	13.6	7.6
FNvsWL	8.2	22.0	1.6	13.7	1.5	8.2	13.3	9.8	9.9	11.7
MAvsWN	10.8	12.4	2.2	4.2	4.8	10.8	12.4	12.9	16.9	12.6
SNvsWL	9.0	2.2	1.8	7.7	0.8	9.0	2.2	32.2	32.2	3.0
SNvsWN	11.2	12.2	2.2	3.4	4.7	11.2	12.2	15.4	15.4	12.0
Entire network	12.1	12.3	2.0	7.7	2.9	14.3	9.7	16.5	14.4	8.1
Included studies	2	1	5	1	2	2	2	3	1	1

FUNCTION

Direct comparisons in the network (columns); Network meta-analysis estimates (rows)

	EAvsEDU	EAvsFN	EAvsSN	EAvsWL	EAvsWN	EDUvsSN	FNvsWN	MAvsSN	MAvsWL	WLvsWN
**Mixed estimates**										
EAvsEDU	10.3	[illegible]	4.7	12.9	1.5	22.6	3.5	18.0	18.0	5.0
EAvsFN	0.4	58.3	1.3	7.1	5.7	0.4	14.6	1.7	1.7	8.8
EAvsSN	1.9	5.1	6.7	18.6	2.1	1.9	5.1	25.8	25.8	7.2
EAvsWL	1.8	9.7	6.6	35.6	4.1	1.8	9.7	8.4	8.4	13.8
EAvsWN	0.7	25.0	2.4	13.2	10.6	0.7	25.0	3.1	3.1	16.3
EDUvsSN	14.6	2.3	3.0	8.4	1.0	42.0	2.3	11.6	11.6	3.3
FNvsWN	0.5	20.0	1.8	9.8	7.9	0.5	42.7	[illegible]	2.3	12.1
MAvsSN	0.3	0.3	1.0	0.9	0.1	0.3	0.3	95.3	[illegible]	0.4
MAvsWL	1.6	1.4	5.7	5.2	0.6	1.6	1.4	7.3	73.1	2.0
WLvsWN	1.0	16.1	3.4	18.5	6.8	1.0	16.1	4.4	4.4	28.4
**Indirect estimates**										
EAvsMA	2.1	6.2	7.7	22.8	2.6	2.1	6.2	9.8	31.6	8.8
EDUvsFN	8.0	17.9	3.2	7.6	0.9	17.9	7.9	14.7	14.7	7.1
EDUvsMA	11.1	1.8	1.8	6.7	0.8	31.2	1.8	33.0	9.2	2.6
EDUvsWL	9.8	2.1	1.0	7.8	0.9	25.1	2.1	24.1	24.1	3.0
EDUvsWN	7.8	9.8	2.5	3.6	4.2	18.3	9.8	15.9	15.9	12.2
FNvsMA	1.5	21.2	5.4	14.8	0.5	1.5	11.4	7.0	25.7	10.9
FNvsSN	1.4	18.1	4.9	12.3	0.4	1.4	9.6	21.4	21.4	9.2
FNvsWL	1.2	27.8	4.2	22.7	0.3	1.2	16.1	5.4	5.4	15.8
MAvsWN	1.3	11.7	4.8	10.4	4.9	1.3	11.7	6.2	29.0	18.6
SNvsWL	1.1	1.0	3.9	3.6	0.4	1.1	1.0	43.2	43.2	1.4
SNvsWN	1.2	9.6	4.3	8.2	4.1	1.2	9.6	23.3	23.3	15.1
Entire network	4.2	12.4	4.0	11.9	2.7	9.2	9.5	17.2	19.2	9.9
Included studies	2	1	5	1	2	2	1	3	1	1

Cumulative contribution plots for the KOA network by all interventions in pain and function.

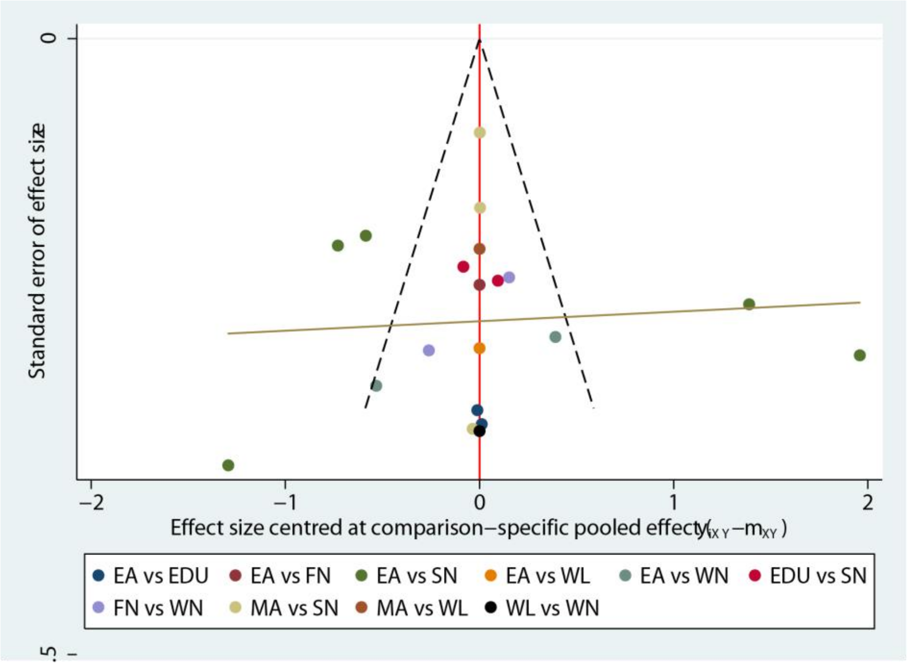

PAIN

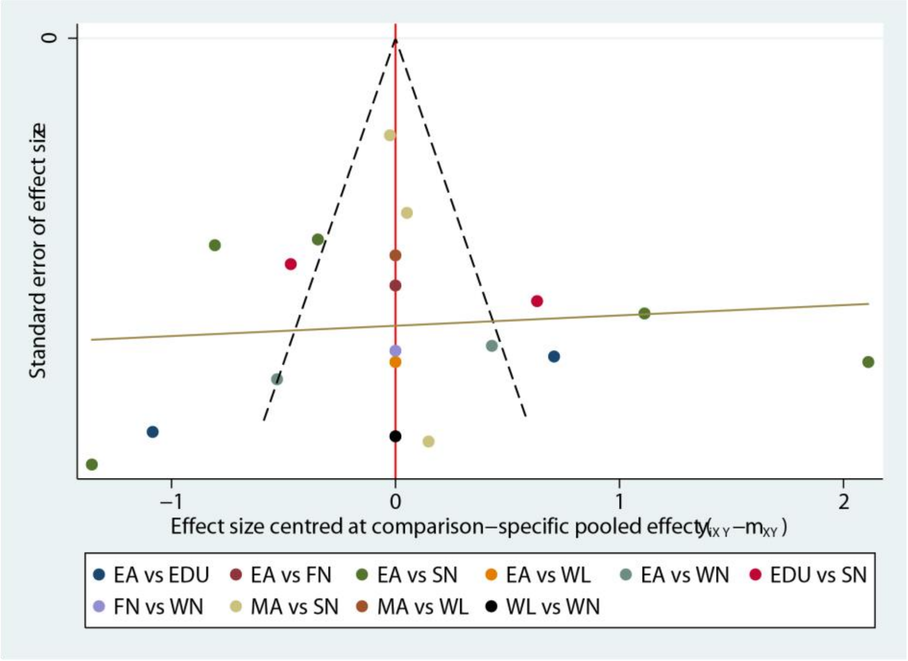

FUNCTION

Funnel plots of all included studies referring to (WOMAC) pain and physical function scores.

Results (MD, with 95% CI) of the pairwise meta-analysis for pain and function scores.

	Sham needle VS Education	Acupuncture VS Sham needle	Electro-acupuncture VS Sham needle	Electro-acupuncture VS Warm needle	Acupuncture VS Waiting list	Electro-acupuncture VS Education	Electro-acupuncture VS Waiting list	Fire needle vs Electro-acupuncture	Fire needle VS Warm needle	Warm needle VS Waiting list
Pain	***-1.14 [-1.20, -1.08]***	***-0.35 [-0.66, -0.03]***	***-1.53 [-1.94, -1.12]***	***-2.35 [-3.92, -0.78]***	***-1.80 [-3.11, -0.49]***	***-2.09 [-2.15, -2.03]***	***-3.01 [-4.71, -1.31]***	***-2.57 [-3.67, -1.47]***	-1.70 [-4.25, 0.85]	***-4.26 [-6.50, -2.02]***
Function	***-3.78 [-3.95, -3.61]***	-0.20 [-0.56, 0.17]	***-4.27 [-5.57, -2.97]***	3.15 [-4.31, 10.61]	***-5.70 [-10.28, -1.12]***	***-6.56 [-6.74, -6.38]***	***-11.98 [-18.01, -5.95]***	***-1.80 [-3.11, -0.49]***	-1.74[-10.26, 6.77]	***-14.70 [-23.86, -5.54]***

Rank probabilities of all treatments with SUCRA values.

**Treatment**	**Pain**	**Rank**	**Function**	**Rank**
Acupuncture	0.54	4	0.62	5
Education	0.78	6	0.75	6
Electro-acupuncture	0.18	2	0.17	2
Fire needle	0.03	1	0.10	1
Sham needle	0.63	5	0.59	4
Waiting list	0.95	7	0.98	7
Warm needle	0.42	3	0.30	3

Sensitivity Analysis of the included studies for pain.

	MD 95%CI	*P*-value	tau^2	I^2
**Sham needle VS Education**				
Omitting Berman 2004	-1.14 [-1.22, -1.06]	< 0.001	0.00	-
Omitting Manheimer 2006	-1.14 [-1.22, -1.06]	< 0.001	0.00	-
**Pooled estimate**	-1.14 [-1.20, -1.08]	< 0.001	0.00	0.00
**Acupuncture VS Sham needle**				
Omitting Chen 2013	-0.10 [-0.47, 0.26]	0.58	0.00	0.00
Omitting Scharf 2006	-0.48 [-0.55, -0.41]	< 0.001	0.00	0.00
Omitting Takeda 1994	-0.33 [-0.70, 0.03]	0.07	0.05	0.75
**Pooled estimate**	-0.35 [-0.66, -0.03]	0.03	0.04	0.52
**Electro-acupuncture VS Sham needle**				
Omitting Berman 2004	-2.25 [-3.42, -1.08]	0.0002	1.05	0.97
Omitting Jubb 2008	-1.52 [-1.94, -1.10]	< 0.001	0.13	0.98
Omitting Sangdee 2002	-0.99 [-1.18, -0.80]	< 0.001	0.02	0.82
Omitting Vas 2004	-1.39 [-1.78, -1.00]	< 0.001	0.12	0.97
Omitting Manheimer 2006	-2.25 [-3.42, -1.08]	0.0002	1.05	0.97
**Pooled estimate**	-1.53 [-1.94, -1.12]	< 0.001	0.13	0.97
**Electro-acupuncture VS Warm needle**				
Omitting Gao 2012	-1.32 [-3.45, 0.81]	0.02	0.00	-
Omitting Lu 2014	-2.97 [-4.36, -1.58]	< 0.001	0.00	-
**Pooled estimate**	-2.35 [-3.92, -0.78]	0.03	0.52	0.65
**Fire needle VS Warm needle**				
Omitting Fan 2016	-3.20 [-5.26, -1.14]	0.002	0.00	-
Omitting Zhang 2013	-0.57 [-1.22, 0.08]	0.08	0.00	-
**Pooled estimate**	-1.70 [-4.25, 0.85]	0.19	2.85	0.82
**Electro-acupuncture VS Education**				
Omitting Berman 2004	-2.09 [-2.17, -2.01]	< 0.001	0.00	-
Omitting Manheimer 2006	-2.09 [-2.17, -2.01]	< 0.001	0.00	-
**Pooled estimate**	-2.09 [-2.15, -2.03]	< 0.001	0.00	0.00

MD: mean difference, CI: confidence interval.

Sensitivity Analysis of the included studies for physical function.

	MD 95%CI	*P*-value	tau^2	I^2
**Sham needle VS Education**				
Omitting Berman 2004	-3.78 [-4.02, -3.54]	< 0.001	0.00	-
Omitting Manheimer 2006	-3.78 [-4.02, -3.54]	< 0.001	0.00	-
**Pooled estimate**	-3.78 [-3.95, -3.61]	< 0.001	0.00	0.00
**Acupuncture VS Sham needle**				
Omitting Chen 2013	-0.20 [-0.57, 0.17]	0.29	0.00	0.00
Omitting Scharf 2006	-0.04 [-3.28, 3.21]	0.98	0.00	0.00
Omitting Takeda 1994	-0.20 [-0.56, 0.17]	0.29	0.00	0.00
**Pooled estimate**	-0.20 [-0.56, 0.17]	0.29	0.00	0.00
**Electro-acupuncture VS Sham needle**				
Omitting Berman 2004	-5.89 [-9.55, -2.22]	0.002	10.28	0.97
Omitting Jubb 2008	-4.42 [-5.74, -3.10]	< 0.001	1.36	0.97
Omitting Sangdee 2002	-2.78 [-3.45, -2.11]	< 0.001	0.23	0.85
Omitting Vas 2004	-3.82 [-5.05, -2.59]	< 0.001	1.16	0.97
Omitting Manheimer 2006	-5.92 [-9.43, -2.41]	0.0009	9.26	0.97
**Pooled estimate**	-4.27 [-5.57, -2.97]	< 0.001	1.35	0.97
**Electro-acupuncture VS warm needle**				
Omitting Gao 2012	-5.60[-10.18,-1.02]	< 0.001	0.00	-
Omitting Lu 2014	3.15 [-4.31, 10.61]	0.41	0.00	-
**Pooled estimate**	-1.74[-10.26, 6.77]	0.40	28.32	0.74
**Electro-acupuncture VS Education**				
Omitting Berman 2004	-6.56 [-6.83, -6.29]	< 0.001	0.00	-
Omitting Manheimer 2006	-6.56 [-6.80, -6.32]	< 0.001	0.00	-
**Pooled estimate**	-6.56 [-6.74, -6.38]	< 0.001	0.00	0.00

MD: mean difference, CI: confidence interval.
